# Rima Glottidis Closed by Warty Vegetations

**Published:** 1846-04

**Authors:** 


*Rima Glottidis closed by Warty Vegetations.*—The case was related by Dr. Stokes, at the Dublin Pathological Society. The patient was a house-painter, aged 34, admitted into the Meath Hospital. He had colica-pictonum thirteen times, and his upper extremities had been paralysed three times. His voice had gradually become very weak, and about eight months ago, he became troubled with cough and expectoration of a viscid mucus, but had neither hæmoptysis nor pain in the chest. At the time of his admission he was extremely emaciated; his countenance pale; lips blue; mouth, teeth, and tongue covered with sordes. His aspect was altogether that of a person suffering from profound disease, and whose constitution had completely given way. There was difficulty of breathing. On looking into the throat, nothing could be perceived but general relaxation. The uvula appeared enlarged, but there was neither ulceration nor cicatrix in the throat. The epiglottis felt healthy, and was not enlarged. There was no dysphagia, and no tumour in the neck. The respiration was sometimes difficult, sometimes easy and tranquil; there was never orthopnœa. There was occasionally stridor, but it was very slight, and only occurred when the patient was excited. The voice was very weak. The respiratory murmur was almost inaudible; sometimes some slight bronchial rales would be heard in the chest; sometimes a very slight degree of dulness might be perceived. The impulse of the heart was well defined, and the sounds normal. When the respiration was suspended by voluntary effort, the first sound of the heart became inaudible. The difficulty of breathing increased, but there were no violent paroxysms of it. The stridor would, perhaps, accompany three or four inspirations, and then intermit. At night there was some slight aberration of mind, but when roused he was perfectly intelligent. He had been despaired of from the time of his admission, and died in about a week afterwards. After death the body was examined. It was then found that the glottis was completely obstructed by a growth of warty vegetations round the lips of the orifice, which was so perfectly closed, that water would not pass through into the larynx when poured upon it. There was nothing else of consequence.

There had been a case some time ago in the Richmond Hospital, the symptoms of which were great dyspnœa, occurring in paroxysms, with stridulous breathing and raucous voice. In that case the obstruction of the larynx depended on the growth of excrescences about the glottis; it was connected with syphilis, and was relieved by tracheotomy. In the present case there had never been any syphilitic affection.—*Dub. Journ.*, March, 1844.

